# Sex-Based Differences in pH Parameters and Esophageal Impedance of Patients With Gastroesophageal Reflux Disease

**DOI:** 10.3389/fmed.2021.629302

**Published:** 2021-05-28

**Authors:** Bixing Ye, Yanjuan Wang, Lin Lin, Liuqin Jiang, Meifeng Wang

**Affiliations:** Department of Gastroenterology, The First Affiliated Hospital of Nanjing Medical University, Nanjing, China

**Keywords:** gastroesophageal reflux disease, sex, reflux episode, mean nocturnal baseline impedance, post-reflux swallow-induced peristaltic wave index

## Abstract

**Background/Aims:** The incidence of reflux esophagitis (RE) has a striking predominance in males. Conversely, non-erosive reflux disease (NERD) is more common in females. This imbalance of gastroesophageal reflux disease (GERD) implies sex-related differences in its pathogenesis. However, limited studies have analyzed the sex-based differences in pH parameters and esophageal impedance of GERD patients.

**Methods:** This study evaluated sex-based pathogenesis differences by comparing reflux episodes, mean nocturnal baseline impedance (MNBI) values, and post-reflux swallow-induced peristaltic wave (PSPW) index values of males with GERD and females with GERD using 24-h multichannel intraluminal impedance and pH monitoring.

**Results:** We analyzed 181 patients (102 males and 79 females) with GERD. Reflux symptom index (RSI) scores were higher in females than that in males (*P* < 0.05). Males had significantly longer acid exposure times, higher DeMeester scores, and more acid reflux episodes than females (*P* < 0.05). Females had more instances of weakly acidic reflux than males (*P* < 0.01). The PSPW index values of males and females were similar (*P* > 0.05). Compared with females, males had lower MNBI values for the mid and distal esophagus (*P* < 0.05). However, with increasing age, the MNBI values of females decreased more rapidly than those of males. MNBI values of elderly patients of both sexes older than 60 years were similar.

**Conclusions:** Acid reflux is more likely to occur in males; however, females tend to have more instances of weakly acid reflux. The integrity of the esophageal mucosa is more fragile in males than in females; however, the esophageal mucosal barrier attenuates more rapidly with increasing age in females than in males.

## Introduction

Gastroesophageal reflux disease (GERD) is a common gastrointestinal disease that affects all age groups and both sexes, with an estimated worldwide prevalence of 8–33% (1). Most epidemiologic studies have suggested that the incidence of reflux esophagitis (RE) has a striking predominance in males (2–5). The male-to-female ratio of the RE prevalence is 1.2–1.6 (2–5). Moreover, the male-to-female ratios of Barrett's esophagus (BE) and esophageal adenocarcinoma (EAC) are approximately 2:1 and 3–5:1 (3, 4). Conversely, non-erosive reflux disease (NERD) and symptomatic GERD are more common in females (5, 6). The sex-based imbalance of GERD implies sex-related differences in its pathogenesis.

It is widely accepted that the vital GERD mechanism is an increase in offensive factors (excessive esophageal noxious content exposure) and defects of defensive factors (mucosa injury, impaired esophageal peristalsis, and damaged anti-reflux barrier). Previous studies have suggested that females have less distal esophageal acid exposure than male healthy volunteers and males with GERD (7, 8). However, most studies have demonstrated that non-acid reflux, namely weakly acidic reflux and weakly alkaline reflux, also result in esophageal mucosal damage (9, 10) and are refractory to proton pump inhibitor (PPI) therapy (11, 12). However, the differences in non-acid reflux of males and females with GERD are still ambiguous.

One study reported that the degree of macroscopic damage observed during endoscopy and the manifestation of RE were more severe in males than in females. In fact, dilated intercellular spaces (DIS), which are signs of mucosal injury, have been observed in NERD patients (10, 13). However, in NERD patients, it is unclear whether there are similar sex-based differences in mucosa injury. The mean nocturnal baseline impedance (MNBI) is a novel impedance parameter measured by 24-h esophageal multichannel intraluminal impedance and pH (MII-pH) monitoring. MNBI represents the permeability of the esophageal mucosa. A negative correlation between MNBI values and intercellular spaces in the esophagus has been observed (14, 15). Previous studies indicated that MNBI values were lower in RE and NERD patients than in reflux hypersensitivity (RH) patients, functional heartburn (FH) patients, and healthy individuals (14, 16, 17). Low MNBI values reflect reflux-stimulated mucosal impairment, even in the absence of macroscopic damage (18). Therefore, it is possible to use MNBI to study sex-based differences in mucosal impairment in RE and NERD.

The post-reflux swallow-induced peristaltic wave (PSPW) index, which is another novel impedance parameter detected by 24-h MII-pH monitoring, reflects the reflux-induced chemical clearance (primary peristalsis). Chemical clearance consists of a salivary swallow elicited by an esophago-salivary vagal reflex and delivery of bicarbonate and epidermal growth factor, thereby augmenting the esophageal pH and hastening repair of reflux-induced mucosal damage. The PSPW index has been reported to efficiently separate GERD patients from healthy individuals (19). Accordingly, the PSPW index was significantly lower in PPI-refractory RE than in healed RE and in PPI-refractory NERD, thus implying that impairment of chemical clearance has a role in the mechanism of reflux-induced esophageal mucosal damage (20). Therefore, a comparison of the PSPW index values of males and females might contribute to clarifying sex-related differences in pathogenesis.

Most studies have focused on sex-based differences in GERD prevalence, symptoms, and response to PPI; however, studies of sex-based differences in pH parameters and esophageal impedance are rare. Therefore, this study aimed to clarify the different pathogenesis based on sex by comparing reflux episodes, MNBI, and the PSPW index values of males with GERD and females with GERD using 24-h MII-pH monitoring.

## Methods

### Subjects

Consecutive patients with GERD symptoms who underwent 24-h MII-pH monitoring at the Gastrointestinal Motility Center of the First Affiliated Hospital of Nanjing Medical University between January 2011 and December 2019 were retrospectively reviewed during our study. Within 3 months before MII-pH monitoring, patients underwent upper endoscopy to evaluate the esophageal mucosal macroscopic status and exclude other upper gastrointestinal disorders. The severity of RE was graded using the Los Angeles (LA) classification. The baseline symptoms were assessed using the Reflux Disease Questionnaire (RDQ) and Reflux Symptom Index (RSI). The inclusion criteria were as follows: age 18 years or older; symptoms of GERD at least two times per week for the past 6 months; and fulfilled the diagnostic criteria for GERD according to the Lyon Consensus (21) [distal esophageal acid exposure time (AET) > 6% on pH impedance monitoring with or without mucosal damage observed during upper endoscopy]. The exclusion criteria were as follows: tumor, peptic ulcer, or other organic lesions observed during endoscopy; history of gastrointestinal surgery; severe organ dysfunction or esophageal motility disorder; and PPI or any antacid medication within 7 days before MII-pH monitoring. The study was approved by the ethics committee of the First Affiliated Hospital of Nanjing Medical University, and all patients provided written informed consent.

### MII-pH Monitoring for 24 h

The esophageal intraluminal impedance and pH values were measured using an ambulatory MII-pH monitoring system (Given Imaging, Duluth, GA). After calibration in pH 4.0 and pH 7.0 buffer solutions, the MII-pH probe was positioned in the esophageal body with the pH sensor at 5 cm and six intraluminal impedance channels (Z1, Z2, Z3, Z4, Z5, and Z6, respectively) at 17, 15, 9, 7, 5, and 3 cm above the lower esophageal sphincter (LES). During the 24-h MII-pH monitoring test, postures, meals, and symptoms were recorded by pressing a button on the data recorder. The impedance-pH tracings were manually assessed by two researchers using the Bioview analysis software program.

### Data Analysis

#### Reflux Episodes

The pH parameters, including DeMeester score, AET, number of acid reflux episodes, number of prolonged reflux episodes, and duration of the longest reflux episodes, were analyzed. AET was defined as the total time when pH was < 4 in the distal esophagus divided by the total duration of MII-pH monitoring; pH < 4 for more than 5 min indicated a prolonged reflux episode.

The impedance parameters of liquid and mixed (liquid-gas) reflux episodes were measured to determine acid (nadir pH < 4), weakly acidic (nadir pH between 4 and 7), and weakly alkaline reflux (nadir pH > 7). Reflux episodes were considered proximal when they extended into or above the channel 15 cm above the LES.

### Post-reflux Swallow-Induced Peristaltic Wave Index

According to a previous study (17), PSPW was defined as an antegrade 50% decrease in impedance occurring within 30 s after a reflux event originating in the most proximal impedance channels and reaching the most distal impedance channel, followed by at least 50% return to the baseline. The PSPW index value was obtained by dividing the number of PSPW by the number of reflux events.

### Mean Nocturnal Baseline Impedance

MNBI (expressed in ohms) was measured during the night recumbent period at three time points (~1:00 a.m., 2:00 a.m., and 3:00 a.m.) during three 10-min periods to avoid reflux and swallowing. The MNBI was calculated using the three impedance values (22). The proximal, mid, and distal MNBI values were measured at 17 and 15 cm above the LES (Z1 and Z2), at 9 and 7 cm above the LES (Z3 and Z4), and at 5 and 3 cm above the LES (Z5 and Z6), respectively (23).

### Statistical Analysis

Normality of the continuous variables was assessed using the Kolmogorov–Smirnov test or Shapiro–Wilk test, depending on the sample size. If the continuous variables were normally distributed, then they were expressed as mean ± standard deviation (SD) and compared using the two-tailed Student *t*-test. Otherwise, data were expressed as medians and interquartile ranges (IQR; 25th−75th percentile) and compared using the Mann–Whitney U test. Categorical variables were compared using the χ2 test or Fisher's exact test. Correlation between MNBI and age, pH-impedance parameters, PSPW index were performed with Pearson's correlation coefficient (two-tailed). Multivariate analyses were performed using linear regression for MNBI values as a dependent variable and the associations of sex, age, reflux episodes, and PSPW index as appropriate. Non-standardized coefficients (β) and 95% confidence intervals (CI) for each of the variables examined were calculated. Because age is associated with the MNBI, the interaction effects of sex and age on MNBI values were analyzed using a two-way analysis of variance (ANOVA). *P* < 0.05 was considered statistically significant. All data were analyzed using SPSS (version 20; IBM Corp., Armonk, NY) and Prism software (version 8; Graph Pad, San Diego, CA).

## Results

### Demographics and Clinical Characteristics

A total of 699 patients were referred for 24-h MII-pH monitoring at our hospital from 2011 to 2019. Among them, 181 patients were finally enrolled in our study, 518 patients were excluded (503 patients with AET < 6%, six patients with cardiac surgery, four patients with peptic ulcer, three patients with achalasia, and two patients with lung transplantation). In 181 GERD patients, there were 102 (56.4%) males and 79 (43.6%) females with ages ranging from 21 to 78 years (mean age, 51.1 ± 12.0 years). Endoscopy revealed that 56 patients had erosive esophageal mucosa (40 with LA grade A and 16 with LA grade B). A total of 120 (66.3%) patients presented with typical symptoms (73 with heartburn and 47 with regurgitation) and 61 presented with atypical symptoms (33 with cough, 16 with chest pain, four with abdominal discomfort, four with belching, and four with dysphagia). RSI scores were higher in females [6.0 *IQR* (4.0–9.0)] than that in males [4.0 *IQR* (4.0–8.0)], *P* = 0.016. There were no statistically significant differences in age, body mass index (BMI), RE, and RDQ scores of males and females (Table 1).

**Table 1:** Demographics and clinical characteristics according to sex.

	**Males (*n =* 102)**	**Females (*n =* 79)**	***P*-value**
Age (years)	49.7 ± 12.3	52.9 ± 11.4	0.07
Age groups (years)			0.17
≤40	26 (25.5%)	11 (13.9%)	
41–50	25 (24.5%)	19 (24.1%)	
51–60	33 (32.4%)	27 (34.2%)	
>60	18 (17.6%)	22 (27.8%)	
BMI, kg/m^2^	23.7 ± 3.6	23.2 ± 2.9	0.28
RE, *n* (%)	36 (35.3%)	20 (25.3%)	0.19
Typical symptoms, *n* (%)	73 (71.6%)	47 (59.5%)	0.11
RDQ	10.0 (7.0–14.0)	8.0 (0–8.0)	0.24
RSI	4.0 (4.0–8.0)	6.0 (4.0–9.0)	0.016

*Data are presented as mean ± SD, number (percentage) or the median and interquartile range. BMI, body mass index; RE, reflux esophagitis; RDQ, Reflux Disease Questionnaire; RSI, Reflux Symptom Index*.

### Reflux Episodes

When considering sex and pH parameters, males had significantly longer AET (*P* = 0.033) and higher DeMeester scores (*P* = 0.03) than those of females. More frequent acid reflux episodes, more prolonged acid reflux episodes, and the longest acid reflux episodes occurred in males than in females; however, the differences did not reach significance.

When sex and impedance parameters were examined, males had significantly more acid reflux in the proximal (*P* = 0.01) and distal esophagus (*P* = 0.001). In contrast, females had more instances of weakly acidic reflux in the distal esophagus (*P* = 0.006) than males. There were no significant differences in the number of weakly alkaline reflux episodes in the proximal and distal esophagus and the number of weakly acidic in the proximal esophagus in males and females. The reflux characteristics according to sex are shown in Table 2.

**Table 2:** Reflux characteristics according to sex.

	**Males (*n =* 102)**	**Females (*n =* 79)**	***P*-value**
**pH PARAMETERS**			
AET (%)	8.3 (5.7–13.6)	6.7 (1.8–13.0)	0.033
Acid reflux episodes	95.5 (69.0–187.0)	87.0 (54.0–134.0)	0.051
Prolonged acid reflux episodes	4.6 (2.0–8.3)	3.0 (0–7.0)	0.12
Longest reflux episode (min)	17.5 (8.0–33.0)	11.0 (5.0–32.0)	0.13
DeMeester score	29.6 (22.2–49.6)	23.1 (10–47)	0.03
**IMPEDANCE PARAMETERS**			
Distal extent, total	66.5 (41.0–96.3)	85.0 (43.0–117.0)	0.062
Distal extent, acid	36 (17.8–52.0)	27.0 (12.0–41.0)	0.01
Distal extent, weakly acid	22 (7.8–49.0)	43.0 (14.0–79.0)	0.006
Distal extent, weakly alkaline	0 (0–2.0)	0 (0–3.0)	0.155
Proximal extent, total	44.0 (19.8–78.9)	38.9 (19.9–53.9)	0.19
Proximal extent, acid	22.5 (11.8–40.0)	14.9 (6.0–25.0)	0.001
Proximal extent, weakly acid	11.9 (4.0–31.2)	15.0 (5.0–31.0)	0.493
Proximal extent, weakly alkaline	0 (0–1.0)	0 (0–1.0)	0.65

*Data are presented as the median and interquartile range. AET, acid exposure time*.

No statistically significant differences in AET, DeMeester scores, number of acid reflux episodes in the distal and proximal esophagus, and number of weakly acidic reflux episodes in the distal esophagus among different age groups were observed (all *P* > 0.05) (Table 3).

**Table 3:** Reflux characteristics according to age.

	**40 years or younger (*n =* 37)**	**41–50 years (*n =* 44)**	**51–60 years (*n =* 60)**	**Older than 60 years (*n =* 44)**	***P*-value**
AET (%)	6.3 (3.5–10.2)	5.7 (1.2–13.3)	7.0 (3.2–12.6)	8.4 (6.4–15.6)	0.088
DeMeester score	24.2 (14.2–40.8)	22.6 (7.4–46.5)	26.2 (13.1–47.2)	33.0 (23.1–62.1)	0.105
Distal extent, acid	36.0 (23.0–54.0)	28.5 (15.0–49.5)	33.0 (17.0–46.5)	24.0 (12.0–50.0)	0.198
Distal extent, weakly acid	45.0 (17.0–77.0)	45.5 (16.0–87.0)	38.0 (15.5–38.0)	24.0 (11.0–33.0)	0.153
Proximal extent, acid	26.0 (15.0–42.0)	18.0 (10.9–40.7)	19.9 (8.9–31.9)	16.9 (8.9–26.0)	0.233

*Data are presented as the median and interquartile range. AET, acid exposure time*.

### Post-reflux Swallow-Induced Peristaltic Wave Index and Mean Nocturnal Baseline Impedance

There was no significant difference in the PSPW index of males [13.5 *IQR* (6.1–23.8)] and females [11.7 *IQR* (3.4–21.6); *P* = 0.37].

**Figure 1:**
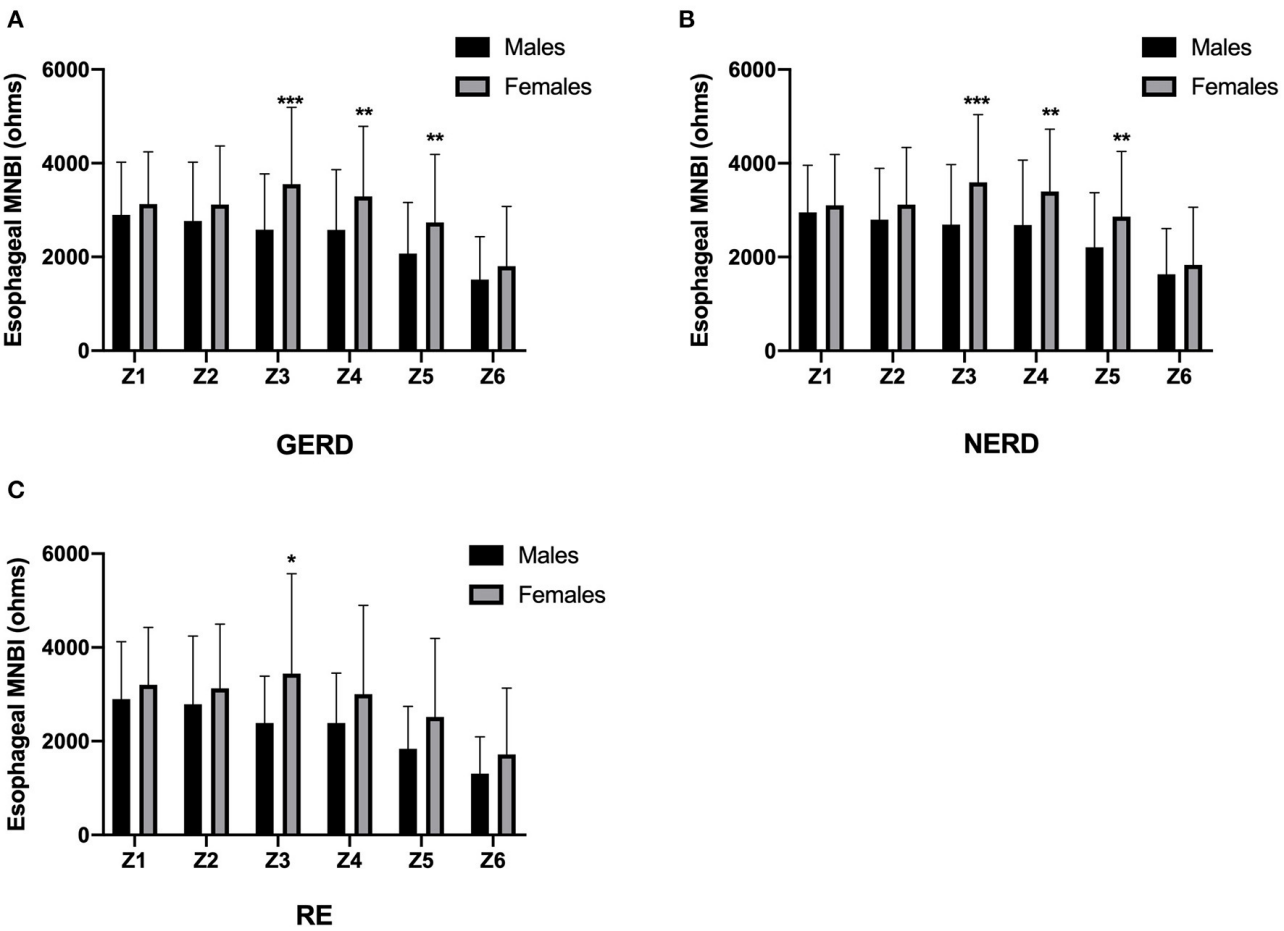
MNBI values from each channel in females and males. MNBI from Z3 to Z5 were lower in males than in females in GERD patients **(A)** and NERD patients **(B)**, MNBI at Z3 were lower in males than in females in RE patients **(C)**. GERD, gastroesophageal reflux disease; NERD, non-erosive reflux disease; RE, reflux esophagitis; MNBI, mean nocturnal baseline impedance. *P < 0.05, **P < 0.01 and *** *P* < 0.001 for males and females in each channel.

All of the MNBI values of six impedance channels were lower in males than in females in GERD, NERD and RE patients, but only MNBI values from Z3 to Z5 in GERD patients (2,582.6 ± 1,189.4 ohms vs. 3,554.7 ± 1,635.7 ohms, *P* < 0.001; 2,576.9 ± 1,283.3 ohms vs. 3,292.2 ± 1,497.7 ohms, *P* = 0.001; 2,074.3 ± 1,088.3 ohms vs. 2,737.1 ± 1,448.7 ohms, *P* = 0.001), MNBI values from Z3 to Z5 in NERD patients (2,691.5 ± 1,279.5 ohms vs. 3,594.6 ± 1,442.7 ohms, *P* < 0.001; 2,682.7 ± 1,387.0 ohms vs. 3,396.5 ± 1,332.4 ohms, *P* = 0.005; 2,205.6 ± 1,164.4 ohms vs. 2,860.5 ± 1,391.9 ohms, *P* = 0.007), and MNBI at Z3 in RE patients (2,389.8 ± 998.9 ohms vs. 3,441.0 ± 2,132.7 ohms, *P* = 0.049) achieved statistical significance (Figure 1).

**Figure 2:**
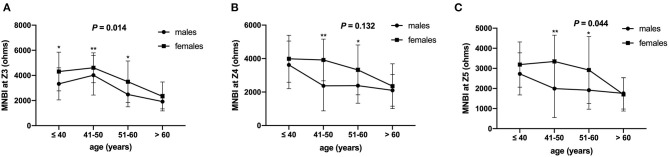
MNBI values at different ages based on sex. MNBI values decreased with increasing age in both males and females, and an interaction effect was observed for age and sex with MNBI values at Z3 **(A)** and Z5 **(C)**, but not at Z4 **(B)**. MNBI, mean nocturnal baseline impedance. *P < 0.05 and **P < 0.01 for males and females in each age group.

In addition, MNBI form Z3 to Z5 showed significant correlations with age, AET, prolonged acid reflux episodes, the longest reflux episode, DeMeester score, total reflux in the distal esophagus, weakly acid reflux in the distal esophagus, total reflux in the proximal esophagus and weakly acid reflux in the proximal esophagus (all *P* < 0.05). The MNBI values associations of age, reflux episodes, and PSPW index are shown in Table 4. After adjusting for above parameters that influenced MNBI values, there were still significant sex-based differences in MNBI values at Z3, Z4, and Z5 (*P* < 0.05). Additionally, age was associated with MNBI values at Z3, Z4, and Z5 (*P* < 0.05). Multivariate analyses of sex, age, and reflux episodes on MNBI are shown in Table 5. Therefore, the interaction of age and sex with MNBI values was further evaluated. The MNBI values decreased with increasing age in both males and females; however, this decreasing trend occurred more quickly in females than in males. Moreover, an interaction effect was observed for age older than 60 years and sex for MNBI values at Z3 and Z5. MNBI values at Z3 and Z5 in elderly patients older than 60 years were similar for both sexes (*P* = 0.014 and 0.044). A more rapid decrease in MNBI values with increasing age occurred in females (Figure 2).

**Table 4:** MNBI values associations of age, reflux episodes, and PSPW index.

	**Z3 (ohms)**	**Z4 (ohms)**	**Z5 (ohms)**
Age (years)	−0.392^***^	−0.373^***^	−0.286^***^
AET (%)	−0.383^***^	−0.444^***^	−0.465^**^
Acid reflux episodes	−0.124	−0.173^*^	−0.227^**^
Prolonged acid reflux episodes	−0.331^***^	−0.346^***^	−0.368^***^
Longest reflux episode (min)	−0.282^***^	−0.324^***^	−0.353^***^
DeMeester score	−0.362^***^	−0.427^***^	−0.460^***^
Distal extent, total	−0.383^**^	−0.385^**^	−0.366^**^
Distal extent, acid	0.028	0.021	−0.015
Distal extent, weakly acid	0.413^***^	0.406^***^	0.409^***^
Distal extent, weakly alkaline	0.111	0.127	0.104
Proximal extent, total	0.190^*^	0.213^**^	0.235^**^
Proximal extent, acid	−0.007	−0.018	−0.049
Proximal extent, weakly acid	0.276^***^	0.317^***^	0.371^***^
Proximal extent, weakly alkaline	0.096	0.107	0.130
PSPW index	0.073	0.086	0.084

*Pearson's correlation coefficient, r. P-values were calculated using Pearson's correlation analysis*.

* *P < 0.05*,

** *P < 0.01 and*

*** *P < 0.001 were considered statistically significant*.

**Table 5:** Multivariate analyses of sex, age, and reflux episodes on MNBI.

	**Z3**		**Z4**		**Z5**	
	**β (95% CI)**	***P***	**β (95% CI)**	***P***	**β (95% CI)**	***P***
Sex	920.7 (547.6–1,293.8)	<0.001	588.5 (220.1–956.9)	0.002	544.9 (199.4–9,890.4)	0.002
Age	−46.1 (−61.1–−31.1)	<0.001	−40.3 (−55.3–−25.3)	<0.001	−26.9 (−40.9–−12.8)	<0.001
AET	−40.9 (−90.5–8.6)	0.105	−50.9 (−99.8–−2.1)	0.041	−31.6 (−77.4–−14.1)	0.174
Prolonged acid reflux episodes	−6.2 (−31.1–18.5)	0.619	2.8 (−21.4–27.1)	0.817	−0.8 (−23.6–22.0)	0.944
Longest reflux episode	−0.83 (−7.9–6.3)	0.817	−0.8 (−7.8–6.2)	0.819	−1.8 (−78.4–4.6)	0.576
DeMeester score	5.1 (−9.5–19.67)	0.490	4.9 (−9.5–19.3)	0.504	0.5 (−13.0–13.9)	0.947
Distal extent, total	3.2 (−8.2–14.6)	0.581	8.3 (−2.9–19.5)	0.147	5.2 (−5.3–15.7)	0.330
Distal extent, weakly acid	3.1 (−11.2–17.4)	0.669	−4.8 (−18.7 −9.3)	0.501	−3.3 (−16.5–9.8)	0.619
Proximal extent, total	−6.2 (−23.1–10.7)	0.468	−16.1 (−32.7–0.5)	0.058	−14.8 (−30.4–0.8)	0.063
Proximal extent, weakly acid	3.4 (−22.1–28.9)	0.793	20.7 (−4.3–0.6)	0.104	25.5 (2.1–49.1)	0.033

*β, non-standardized coefficients; CI, confidence intervals*.

## Discussion

A comprehensive analyses of pH- impedance tracings, including parameters assessing reflux episodes, primary peristalsis, and mucosal integrity of males and females were performed. The main results of this study were as follows: females had higher RSI scores than males; males had significantly longer AET, higher DeMeester scores, and more acid reflux episodes than females; females had more episodes of weakly acidic reflux than males; no statistically significant difference in the PSPW index values of males and females was observed; and males had lower MNBI values at the mid and distal esophagus. However, with increasing age, the MNBI values decreased more rapidly in females than in males.

Many studies have shown a difference in symptom manifestation between females and males with GERD. Previous studies showed that the severity of symptoms in females was significantly more than in males (24), meanwhile, extra-esophageal / atypical symptoms were found to be significantly more common in females than in males (25). Similar with previous studies, our study suggested that RSI scores, reflected severity of laryngopharyngeal reflux, were higher in females than in males. It suggested that females had more severe extra-esophageal symptoms. The visceral hypersensitivity, associated with peripheral sensitization, central sensitization, and psycho-neuroimmune interactions, might cause females to be more susceptible to GERD symptoms than males (3). Further studies are warranted to elucidate the mechanisms responsible for sex and gender differences in symptom perception. Moreover, differential sensitivity and enhanced symptoms in females were regarded to have wide diagnostic and therapeutic implications. For example, it is possible that females present to medical treatment earlier in the course of GRED, and may not develop complications, such as BE and EAC (24).

One study found no difference in the reflux episodes of males and females (24). In contrast, several studies reported that males have higher exposure to distal esophageal acid than female healthy volunteers and females with GERD (7, 8). Our results were consistent with those of previous studies. Additionally, a new finding of our study was that females had more episodes of weakly acid reflux than males. These results partially explained that the esophageal mucosa in males is more fragile than that in females; this was verified by this study. During both *in vitro* and *in vivo* studies (9, 18, 26), acid reflux has triggered more obvious cellular damage of the esophageal mucosa compared with weakly acid reflux. Our previous study verified that distal MNBI values were inversely correlated with acid exposure (27). Although distal MNBI values were lower in the non-acid reflux group than in the non-reflux group, no significant difference was observed (27). However, most studies indicated that females had a poorer response to PPI treatment than males (28–31). The role of non-acid reflux in persistent symptoms in patients with PPI failure has been elucidated. Hence, we speculated that sex-related differences in reflux episodes might cause refractoriness to PPI in females.

The majority of gastroesophageal reflux is removed by volume clearance (secondary peristaltic waves). However, for the complete removal of refluxate, chemical clearance is most often necessary. A recent study (32) showed that the PSPW index was related to the peristaltic reserve of the esophageal smooth muscle evaluated by multiple rapid swallows. Despite conflicting results, several studies reported that esophageal motility was different in male and female healthy participants and male and female GERD patients (33). For instance, one study showed that females had higher LES resting pressures than males (34). On the contrary, other studies reported that no sex-based difference in LES resting pressures were observed (24, 35). In our study, there were no significant sex-based differences in the PSPW index; this had not been studied previously. This result indicated that no sex-based differences in esophagus peristalsis exist; however, more studies are required to confirm this finding.

The predominance of RE in males implied that the esophageal mucosa in males is more vulnerable to refluxed gastroduodenal contents. As expected, our study clarified the lower MNBI values of males with GERD. However, the frequencies of RE and moderate to severe RE increased more rapidly in menopausal females than in males (2, 5); therefore, the incidence of RE for females was similar to that for males at age 90 years (2) or at ages older than 70 years (5). Consistent with these results, our study showed that MNBI values decreased more rapidly in females than in males with increasing age. The decrease in estrogen levels during menopause might have a crucial role in the attenuation of the mucosal barrier. Recent studies of experimental animal models have demonstrated that estrogen protects the esophageal mucosal barrier by anti-inflammatory activity (36), inhibition of oxidative stress (37), and expression of tight junction proteins (38). A few studies have reported that hormone replacement therapy (HRT) administered to menopausal females inhibited the esophageal inflammation of GERD and the risk of EAC (39). However, HRT has been shown to increase the risk of GERD symptoms (40). This result was not applicable to the direct administration of estrogen as a therapeutic reagent for GERD. In the future, several molecules of the estrogen signaling pathway may be a target for menopausal females with PPI-refractory GERD.

There were some limitations to our study. First, this was a retrospective database study. There were no available data regarding the potentially etiological factors of GERD, including drinking alcohol, smoking, *Helicobacter pylori* status, and psychological disorders. If these risk factors were different in females and males, then they might have contributed to the study findings which may be subject to information bias. Second, because the analyzed parameters were reflux episodes, esophageal primary peristalsis, and the mucosal barrier, which are common mechanisms of RE and NERD, we did not stratify GERD into NERD and RE for their respective analyses. However, because the mechanisms of NERD and RE have subtle differences, it might be better to compare sex-based differences in RE and NERD. Third, when we divided the patients into different age groups, the numbers of patients in each group were relatively small; therefore, a statistical bias might have existed.

In conclusion, females had more severe extra-esophageal symptoms, acid reflux is more likely to occur in males, and weakly acid reflux tends to occur more frequently in females. The integrity of the esophageal mucosa is more fragile in males than in females; however, the esophageal mucosal barrier was attenuated more rapidly with increasing age in females than in males. These results offer some evidence that the pathogenesis is different between males and females, and that estrogen is a potentially protective factor of the esophageal mucosa. However, the detailed mechanism of estrogen in controlling the pathogenesis of the GERD spectrum remains to be studied in the future.

## Data Availability Statement

The raw data supporting the conclusions of this article will be made available by the authors, without undue reservation.

## Ethics Statement

The studies involving human participants were reviewed and approved by ethics committee of the First Affiliated Hospital of Nanjing Medical University. Written informed consent to participate in this study was provided by the participants' legal guardian/next of kin. Written informed consent was obtained from the individual(s) for the publication of any potentially identifiable images or data included in this article.

## Author Contributions

BY, YW, LL, and LJ conceptualized the idea for this study. BY and YW performed the analysis and prepared the display items. MW performed esophageal 24-h MII-pH monitoring and analyzed the MII-pH parameters. BY wrote the first draft of the manuscript. LJ revised the manuscript. All authors contributed to the article and approved the submitted version.

## Conflict of Interest

The authors declare that the research was conducted in the absence of any commercial or financial relationships that could be construed as a potential conflict of interest.
